# Effects of Litter and Root Manipulations on Soil Bacterial and Fungal Community Structure and Function in a Schrenk’s Spruce (*Picea schrenkiana*) Forest

**DOI:** 10.3389/fpls.2022.849483

**Published:** 2022-04-14

**Authors:** Haiqiang Zhu, Lu Gong, Yan Luo, Junhu Tang, Zhaolong Ding, Xiaochen Li

**Affiliations:** ^1^College of Ecology and Environment, Xinjiang University, Urumqi, China; ^2^Key Laboratory of Oasis Ecology, Ministry of Education, Urumqi, China; ^3^Ecological Postdoctoral Research Station, Xinjiang University, Urumqi, China

**Keywords:** litter manipulation, soil bacteria, soil fungi, microbial function, Schrenk’s spruce forest

## Abstract

Soil microorganisms are the key driver of the geochemical cycle in forest ecosystem. Changes in litter and roots can affect soil microbial activities and nutrient cycling; however, the impact of this change on soil microbial community composition and function remain unclear. Here, we explored the effects of litter and root manipulations [control (CK), doubled litter input (DL), litter removal (NL), root exclusion (NR), and a combination of litter removal and root exclusion (NI)] on soil bacterial and fungal communities and functional groups during a 2-year field experiment, using illumina HiSeq sequencing coupled with the function prediction platform of PICRUSt and FUNGuild. Our results showed that litter and root removal decreased the diversity of soil bacteria and fungi (AEC, Shannon, and Chao1). The bacterial communities under different treatments were dominated by the phyla Proteobacteria, Acidobacteria, and Actinomycetes, and NL and NR reduced the relative abundance of the first two phyla. For the fungal communities, Basidiomycetes, Ascomycota, and Mortierellomycota were the dominant phyla. DL increased the relative abundance of Basidiomycetes, while NL and NR decreased the relative abundance of Ascomycota. We also found that litter and root manipulations altered the functional groups related to the metabolism of cofactors and vitamins, lipid metabolism, biosynthesis of other secondary metabolites, environmental adaptation, cell growth, and death. The functional groups including ectomycorrhizal, ectomycorrhizal-orchid mycorrhizal root-associated biotrophs and soil saprotrophs in the fungal community were also different among the different treatments. Soil organic carbon (SOC), pH, and soil water content are important factors driving changes in bacterial and fungal communities, respectively. Our results demonstrate that the changes in plant detritus altered the soil microbial community structure and function by affecting soil physicochemical factors, which provides important data for understanding the material cycle of forest ecosystems under global change.

## Introduction

Soil microorganisms are the crucial drivers of the carbon (C) and nitrogen (N) cycles and nutrient dynamics at the plant–soil interface and play a vital role in mediating the biogeochemical cycle of forest ecosystems (Liu et al., 2021). Soil microbes not only control the decomposition of soil C but also affect the transformation process of soil N (i.e., nitrification and denitrification; Crowther et al., 2015; Scarlett et al., 2021). It is well known that environmental changes and human activities can alter the quantity of litter and root inputs into the soil in forest ecosystems (Li et al., 2020; Jing et al., 2021). These alterations can affect the number and community structure of microorganisms by altering soil nutrient availability and hydrothermal factors (Liu et al., 2017; Santiago et al., 2021). Nutrient changes induced by soil microbial changes will in turn affect plant growth and stability in nutrient-deficient forest systems (Zhao et al., 2017). Therefore, exploring the response of forest soil microorganisms to litter and roots is important for accurately evaluating the role of forest soil underground processes in coping with environmental variation.

Soil microorganisms are influenced by the input of exogenous organic matter such as litter and roots, but the magnitude of the effect is closely related to the quantity and quality of organic matter (Fanin and Bertrand, 2016). Previous studies have also shown that the soil microbial community structure has different responses to the input of exogenous organic matter (Habtewold et al., 2020; Veen et al., 2021). For example, litter addition facilitated soil fungal growth and significantly reduced the bacteria: fungi ratio in subtropical forests (Wang et al., 2013). However, in temperate forests, the same treatment had no significant effect on the bacteria: fungi ratio (Brant et al., 2006). Conversely, litter removal (NL) is generally considered to reduce soil microbial biomass and change the ratio of fungi to bacteria (Xu et al., 2013; Pisani et al., 2016), but Yang et al. (2020) showed different trends, indicating that NL has no significant impact on soil microbial community structure. These studies show that soil microbial community composition affected by litter is a complex process that is regulated by multiple factors (Xu et al., 2020). Furthermore, root-derived C input is another important driver of the soil microbial community (Barberan et al., 2015). Several studies have indicated that root-derived C containing recalcitrant chemical components is conducive to the growth of soil fungi, while root exclusion (NR) had an inhibitory effect on the soil fungal community and modified the bacterial community structure (Brant et al., 2006; Sauvadet et al., 2019). Moreover, roots play a more important role in changes in the microbial community and composition than litter (Cantarel et al., 2015). These divergent results showed that changes in litter and roots resulted in a nonlinear relationship with soil microbial community composition, which may be attributed to the differences in the quantity and chemical composition of exogenous substances among different forest ecosystems and the utilization strategies of soil microbial resources (Wang et al., 2019). Accordingly, it is of great priority to thoroughly study the changes and mechanisms of soil microbial community structure change in different forest types.

Soil microbial diversity is closely related to microbial stability, soil quality, and nutrient cycles and is susceptible to the input of exogenous organic matter (Schroeder et al., 2020; Xu et al., 2021). Generally, the increase in resource availability under litter addition treatments contributes to the growth of individual microorganisms (Storch et al., 2018), thus increasing soil microbial diversity (Dilly et al., 2004). NL negatively affects soil microbial diversity by affecting soil microbial stoichiometry and soil moisture (Yang et al., 2020). However, other studies found that litter treatment exerted no significant effect on soil microbial diversity (Zeng et al., 2017). These results may be due to the differences in the proportion of different nutrient types of microorganisms in soil, soil nutrient availability, and hydrothermal factors (Che et al., 2020; Yang et al., 2020). Soil microbial diversity also differs depending on root turnover. Root biomass, decomposition processes, and root exudates can affect soil microbial diversity by changing soil factors such as soil water content, pH, and nutrient availability (Wang et al., 2020a; Wan et al., 2021). Previous studies indicated that roots can have a positive or neutral effect on soil microbial diversity (Wirthner et al., 2011), which may be related to the different physiological tolerances of soil microorganisms in response to soil factor changes (Allison and Martiny, 2008). There have been numerous studies of the response of soil microbiota diversity to litter and root manipulations, but an understanding of the mechanisms underlying soil microbial diversity is still limited (Jing et al., 2021). Therefore, more field experiments are needed to better characterize the response of soil microbial diversity to changes in plant detritus and how these changes interact with the regulation of soil nutrients by changing the output and transformation of C and N.

The changes in soil microbial metabolic functions are closely associated with soil fertility maintenance, material circulation, and the status of the soil system affected by environmental changes (Wang et al., 2020b; Zhou et al., 2020; Han et al., 2021). Previous studies focused primarily on exploring the changes in soil microbial community composition and diversity and its influencing factors but lacked a sufficient understanding of the changes in microbial metabolic functions (Yan et al., 2020). With the development of molecular biology technology, scholars have expanded the focus of attention from soil microbial community composition to functional prediction. Studies have indicated that soil microbial function are closely related to the quantity of plant detritus input into soil (Maillard et al., 2019; Wang et al., 2020b; Feng et al., 2022). For example, Wang et al. (2019) suggested that litter addition can increase the functional groups associated with soil microbial C metabolism, while NL has a negative effect on microbial functional groups. Moreover, root exclusion can alter the osmotic stress genes and functional groups that degrade macromolecule compounds (Shi et al., 2018). Changes in soil microbial function can alter the ability of microorganisms to decompose detritus with high C and N content, which in turn can affect the C and N cycles at the plant–soil interface (Bardgett and van der Putten, 2014). A deeper understanding of the response of soil microbial metabolic functions to plant detritus is crucial for revealing the role of soil microorganisms in the terrestrial biogeochemical cycle.

The Tianshan Mountains harbor the largest montane forest ecosystem in Xinjiang, China, which is highly sensitive to environmental changes (Cowan, 2007). Schrenk’s spruce (*Picea schrenkiana*) account for 90% of the Tianshan forest area and plays a crucial role in conserving water sources, conserving biodiversity and maintaining ecosystem stability. This species exhibits low-quality litter (high C/N ratio) and shallow root systems (Chen et al., 2018), and the influence of this litter and root type on the soil microbial community remains unclear. Thus, we conducted a field experiment to explore how detritus input and removal affect soil microbial (bacteria and fungi) community composition and functional groups in mountainous forest ecosystems in arid areas and to analyze their influencing factors. We hypothesized that (1) litter and root manipulation can significantly change the structure and diversity of soil microbial community and (2) changes in soil nutrients under different treatments drive the shifts in soil microbial community structure and functional groups.

## Materials and Methods

### Study Site

The study site is located in Schrenk’s spruce forest area of Tianshan Mountain, Xinjiang, Northwest China (87.18°E, 43.47°N). Mean annual precipitation is 500 mm and mean annual temperature is 0–4°C (Gong and Zhao, 2019). The dominant species in the region is Schrenk’s spruce. The stand is mostly a pure forest, occurring from mid-mountain to subalpine zone. The understory companion herbs are *Geranium rotundifolium*, *Alchemilla tianschanica*, and *Aegopodium podagraria*. The soil belongs to gray–brown forest soil according to the soil classification of China (China soil systematic classification research cooperation group, 1995).

### Experimental Design and Sample Collection

**Figure 1:**
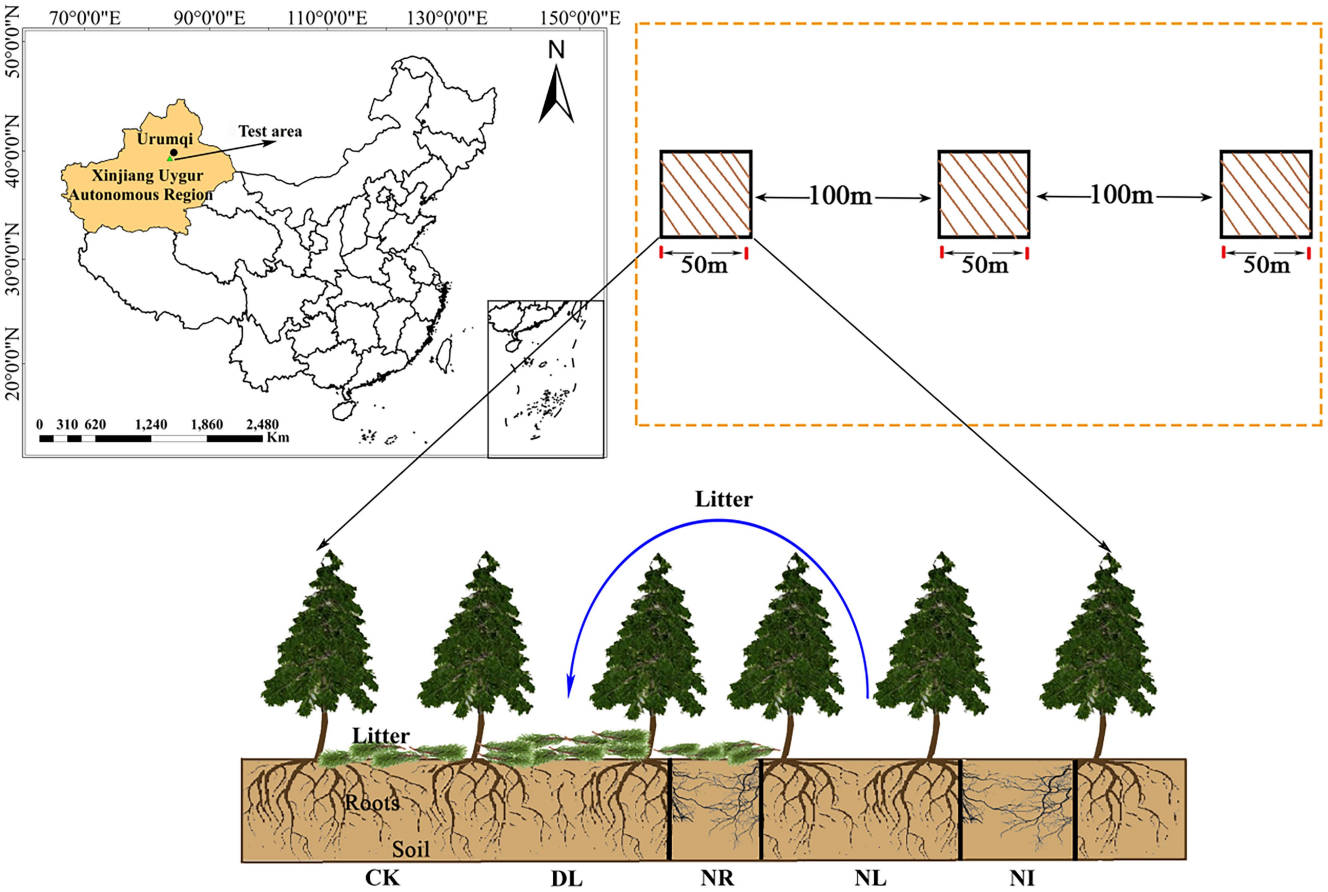
Location of this study and experimental treatments. CK, DL, NL, NR and NI represent control, doubled litter input, litter removal, root exclusion, root and litter exclusion, respectively. Black bars represent PVC boards for excluding roots.

In September 2017, three plots (50 m × 50 m; same altitude, similar tree age and slope) were selected within 1–1.5 m from the trunk. Each plot was divided into five 1 m × 1 m subplots for five treatments: (1) the control (CK), (2) doubled litter inputs (DL), (3) litter removal (NL), (4) root exclusion (NR), and (5) no inputs (NI; Figure 1). For the CK treatment, normal litter inputs were allowed. For the DL treatment, the aboveground litter inputs were doubled by placing litter removed monthly from NL subplots. For the NL treatment, litter was removed with a 0.15-mm nylon mesh suspended 0.5 m above the ground. For the NR treatment, roots were excluded by inserting PVC boards into the trenches (0.1 m wide and 1 m deep). Both the above- and belowground inputs were excluded in the NI subplots.

In September 2019, soil samples from the 0–10 cm soil layer were collected with a soil drill in each subplot. Then, the roots of the companion herbs were removed according to the resilience and toughness of the roots. All soil samples were placed into sterile self-sealing bags and transported to the laboratory. The soil samples were divided into two portions. One portion of the sample was stored at −80°C for Illumina HiSeq sequencing, and the other was dried and then sieved for physicochemical analysis.

### Soil Analysis

#### Soil Property, DNA Isolation, and Illumina HiSeq Sequencing

Soil physicochemical properties were described in our previous studies (Zhu et al., 2021). The DNA from soil bacteria and fungi was extracted following the cetyltrimethylammonium bromide (CTAB) method, and the quality and concentration of DNA were then detected by agarose gel electrophoresis. The primers 515F and 806R were used to amplify the V4 region of the 16S rRNA gene (Peiffer et al., 2013). The ITS1 regions of the fungi were amplified by PCR using the primers ITS5–11737 and ITS2–2043R (Lu et al., 2013). PCR was performed as described below: 1 min of denaturation at 98°C, 30 cycles at 98°C for 10 s, 50°C for 30 s, 72°C for 30 s, and a final extension at 72°C for 5 min. The products obtained after amplification were detected by 2% agarose gel electrophoresis and purified using a GeneJET Gel Extraction Kit (Thermo Scientific, Carlsbad, CA, United States). Following purification, sequencing libraries were generated using an NEB Next® Ultra™ DNA Library Prep Kit for Illumina (NEB, United States) following the manufacturer’s recommendations, and index codes were added. The library was sequenced on an Illumina HiSeq platform at Novogene Biotechnology Co., Ltd., Beijing, China. Then, the raw sequencing data were trimmed and filtered to obtain valid data for subsequent analysis. The sequences were clustered into operational taxonomic units (OTUs) at 97% similarity using Uparse software (Uparse v7.0.1001).^1^ Taxonomic characterization of the representative sequences of bacterial and fungal OTUs were performed using the Silva and Unite databases, respectively. Based on the data after normalization, Chao1, Shannon, and ACE were calculated with QIIME software (version 1.9.1; Caporaso et al., 2010). Additionally, Functional prediction of bacteria was obtained based on the 16S sequencing data using the PICRUSt software. The Kyoto Encyclopedia of Genes and Genomes (KEGG) database was used to annotate functional groups. For fungal communities, the functional groups were predicted using FUNGuild. The fungal OTUs table was uploaded to the FUNGuild platform, and the functional information were predicted by comparing the species OTU with the functional annotation information (Langille et al., 2013; Nguyen et al., 2016). All the Hiseq sequencing data were submitted to the Sequence Read Archive (SRA) of National Center Biotechnology Information (NCBI) database with accession number PRJNA 814000.

### Statistical Analysis

The data analysis was conducted using SPSS 17.0 software (SPSS, IBM, United States). An analysis of variance (ANOVA) with the least significant difference (LSD) was applied to detect significant differences of each variable under different treatments. The differences in soil microbial community composition under different treatments was explored using nonmetric multidimensional scaling (NMDS) based on a Bray-Curtis distance matrix. Adonis analysis was used to compare the significant differences in the soil microbial community composition between different treatments. Redundancy analysis (RDA) and Pearson’s correlation analysis were used to identify the interrelationship between soil bacterial and fungal communities and physicochemical factors. The graphics were created using Origin 2018 (Origin Lab, Massachusetts, United States).

## Results

### Variations in Soil Bacterial and Fungal Community Composition

**Figure 2:**
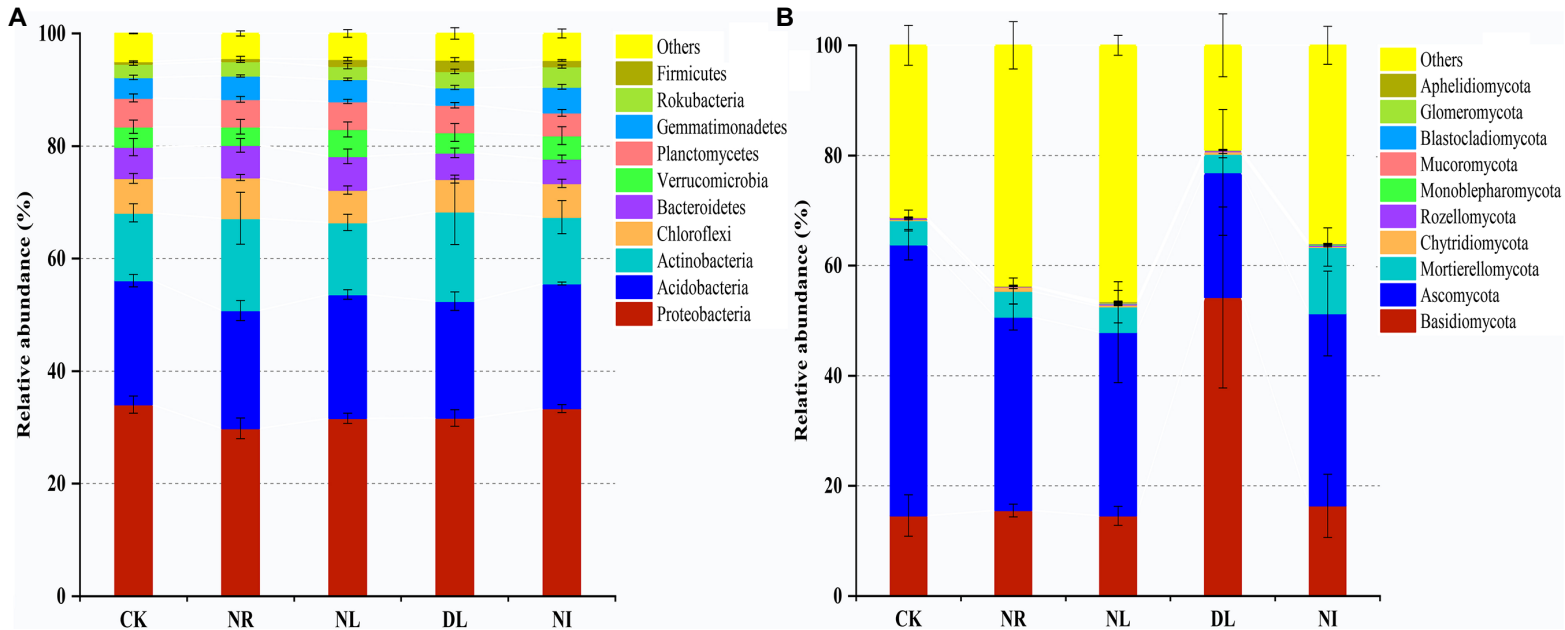
Relative abundance of soil bacteria **(A)** and fungi **(B)** at phylum level under different treatments (*n* = 3). CK, DL, NL, NR, and NI represent control, doubled litter input, litter removal, root exclusion, root and litter exclusion, respectively.

**Figure 3:**
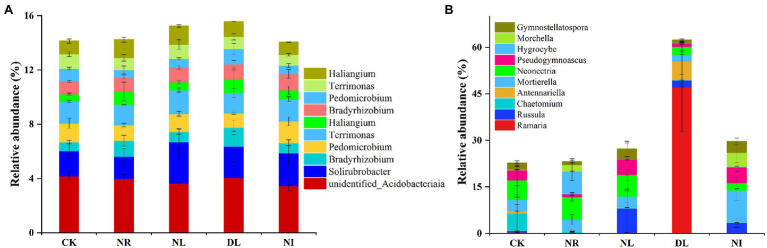
Relative abundance of soil bacteria **(A)** and fungi **(B)** at the genus level under different treatments (*n* = 3). CK, DL, NL, NR, and NI represent control, doubled litter input, litter removal, root exclusion, root and litter exclusion, respectively.

A total of 51 phyla, 58 classes, 121 orders, 206 families, and 423 genera of bacteria were detected in different treated soils (Supplementary Table S1). Proteobacteria (32.10% ± 0.02), Acidobacteria (22.26% ± 0.02), and Actinobacteria (13.79% ± 0.04) were the predominant phyla in the bacterial community (Figure 2A). There were different effects of different treatments on the soil bacterial community (Figure 2A). Compared with the CK, both NL and NR decreased the relative abundance of Proteobacteria and Planctomycetes and increased that of Actinobacteria. The relative abundance of Firmicutes was significantly increased in the DL treatment (*p* < 0.001). At the genus level, NI treatment significantly decreased the relative abundance of unidentified Acidobacteria compared with the CK (*p* < 0.01; Figure 3A). The relative abundance of Haliangium in NL and NI treatments was significantly lower than that in CK (*p* < 0.01). Additionally, the relative abundance of Pedomicrobium was significantly higher in the DL treatment than in NL, NR, and NI treatments (*p* < 0.05; Figure 3A).

A total of 15 phyla, 45 classes, 119 orders, 228 families, and 427 genera of fungi were obtained from soil samples among different treatments (Supplementary Table S1). The most abundant phyla in the fungal community were Basidiomycota (22.80% ± 0.19), Ascomycota (35.05% ± 0.11), and Mortierellomycota (5.83% ± 0.04; Figure 2B). Compared with the CK, the relative abundance of Basidiomycetes increased by 39.62% in the DL treatment (*p* < 0.001). The relative abundance of Ascomycota in NR and NL was significantly lower than that in CK, which was higher by 14.06% and 15.84%, respectively (NR: *p* = 0.05, NL: *p* = 0.03). NI treatment significantly decreased the relative abundance of the phylum Ascomycota while increasing that of Mortierellomycota (Ascomycota: *p* = 0.04, Mortierellomycota: *p* < 0.001). Among the top 10 general, the relative abundance of Ramaria in the DL treatment was significantly higher than that in CK (*p* < 0.001; Figure 3B). Compared with the CK, NI treatment significantly increased the relative abundance of Morchella and Gymnostellatospora (*p* < 0.01). The relative abundance of Antennariella in the DL treatment was significantly higher than that in NL, NR and NI treatments (*p* < 0.05; Figure 3B).

**Figure 4:**
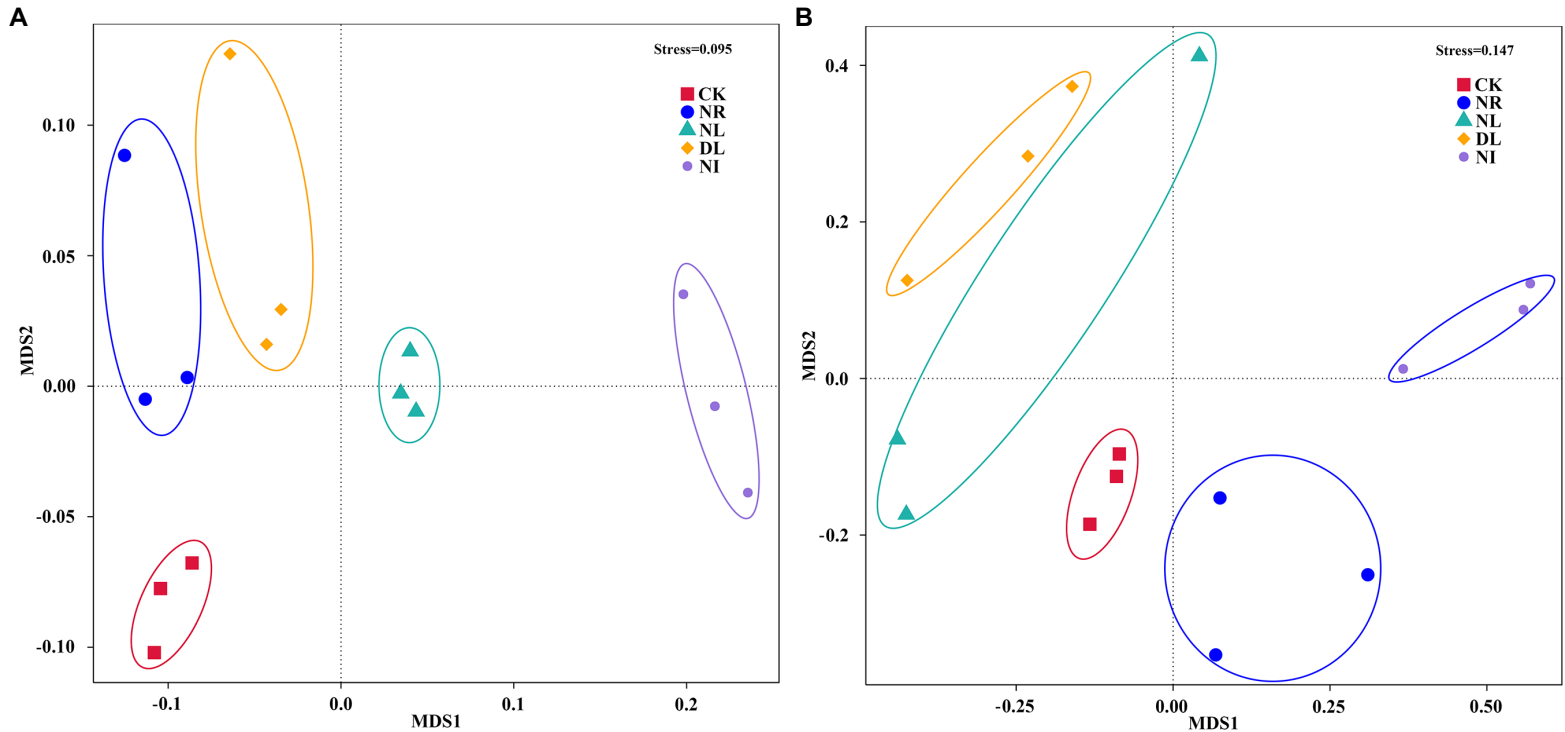
Nonmetric multidimensional scaling (NMDS) of bacterial **(A)** and fungal **(B)** community composition based on Bray–Curtis distances of the OTU matrix. CK, DL, NL, NR, and NI represent control, doubled litter input, litter removal, root exclusion, root and litter exclusion, respectively.

Furthermore, NMDS indicated a clear separation between samples collected from CK and NI treatments (Figure 4). Results from Adonis analysis also showed that the bacterial and fungal community structure of NI-treated soil significantly differed from those in CK treatment (bacteria: *p* = 6.98, *p* < 0.001; fungi: *p* = 5.73, *p* < 0.001).

### Variations of Alpha Diversity of Soil Bacteria and Fungi

**Figure 5:**
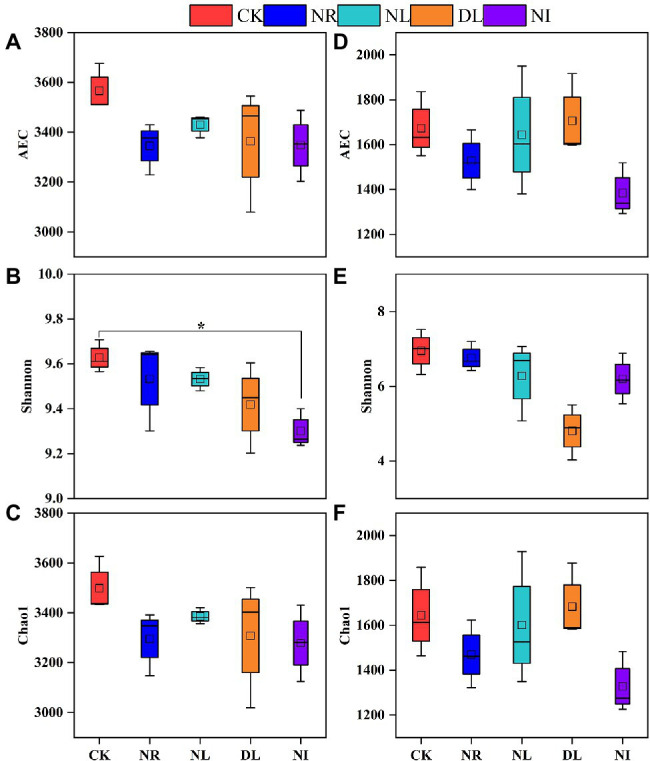
Alpha diversity of soil bacteria **(A–C)** and fungi **(D–F)** under different treatments (*n* = 3). CK, DL, NL, NR, and NI represent control, doubled litter input, litter removal, root exclusion, root and litter exclusion, respectively. Asterisk indicate significance: ^*^p < 0.05.

The observe coverages of soil bacteria and fungi are between 0.987 and 0.992, indicating that the sequencing results can reflect the majority of microbial information (Supplementary Figure S1). Compared to the CK treatment, soil bacterial and fungal diversity (AEC and Chao1 indices) was slightly but not statistically significant decreased in the NL and NR treatments (Figure 5). However, the bacterial Shannon index significantly decreased in the NI treatment (*p* < 0.05; Figure 5). In addition, the decrease in the AEC and Chao1 index of soil bacteria and fungi in the NR treatment was greater than that in the NL treatment.

### Functional Groups of Soil Bacterial and Fungal Communities

**Figure 6:**
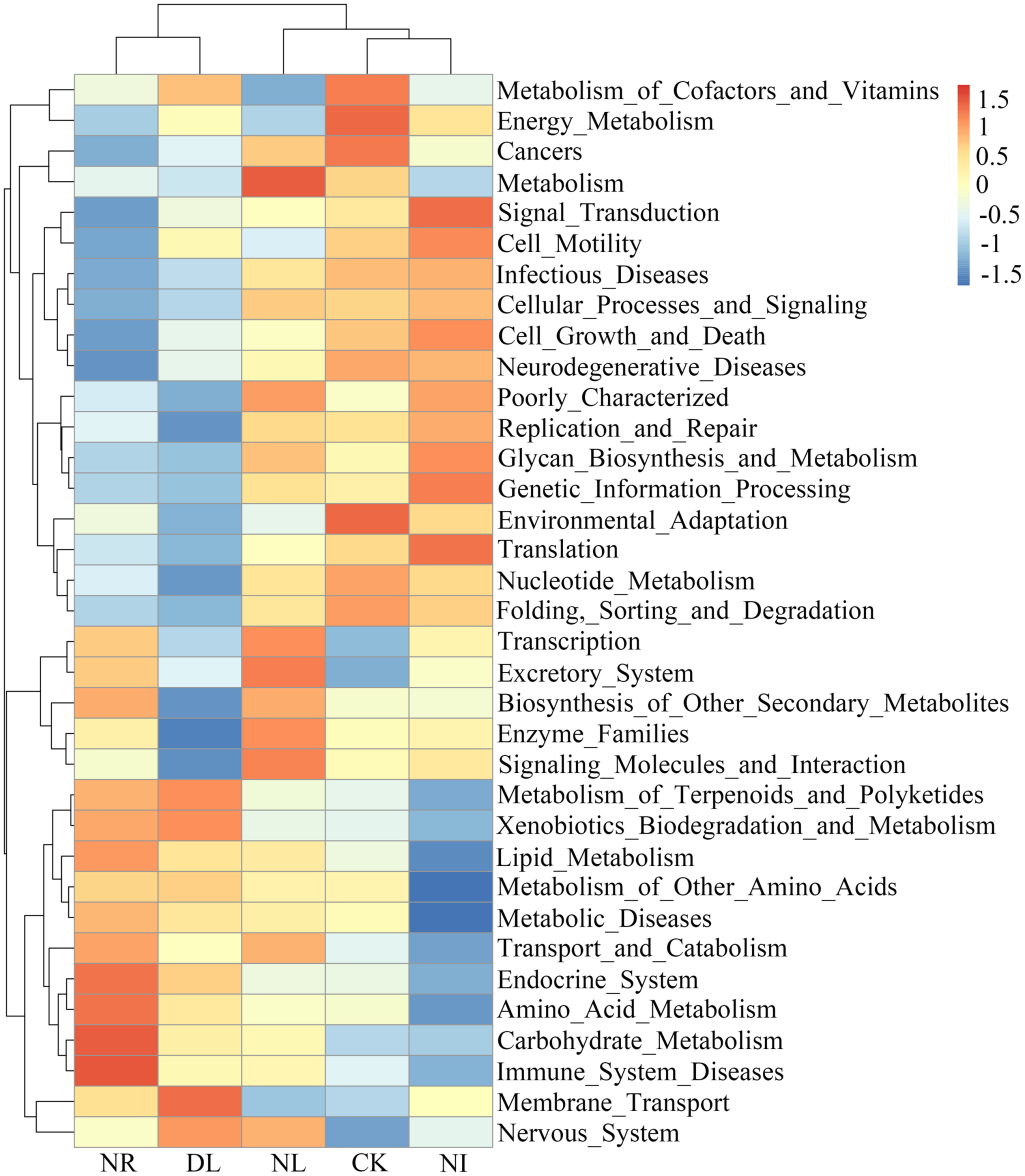
Changes in functional groups of soil bacteria under different treatments. CK, DL, NL, NR, and NI represent control, doubled litter input, litter removal, root exclusion, root and litter exclusion, respectively.

PICRUSt analysis was performed to predict the functional groups of the soil bacterial community. A total of 35 level-two functional groups were obtained in different treatments (Figure 6). The NI treatment significantly reduced the metabolism of cofactors and vitamins compared to the CK treatment (*p* = 0.04). The functional groups associated with the biosynthesis of other secondary metabolites decreased significantly in the DL treatment (*p* = 0.01). Moreover, NL significantly decreased environmental adaptation (*p* = 0.03), metabolism of cofactors and vitamins (*p* = 0.05), and cell growth and death (*p* = 0.03).

**Figure 7:**
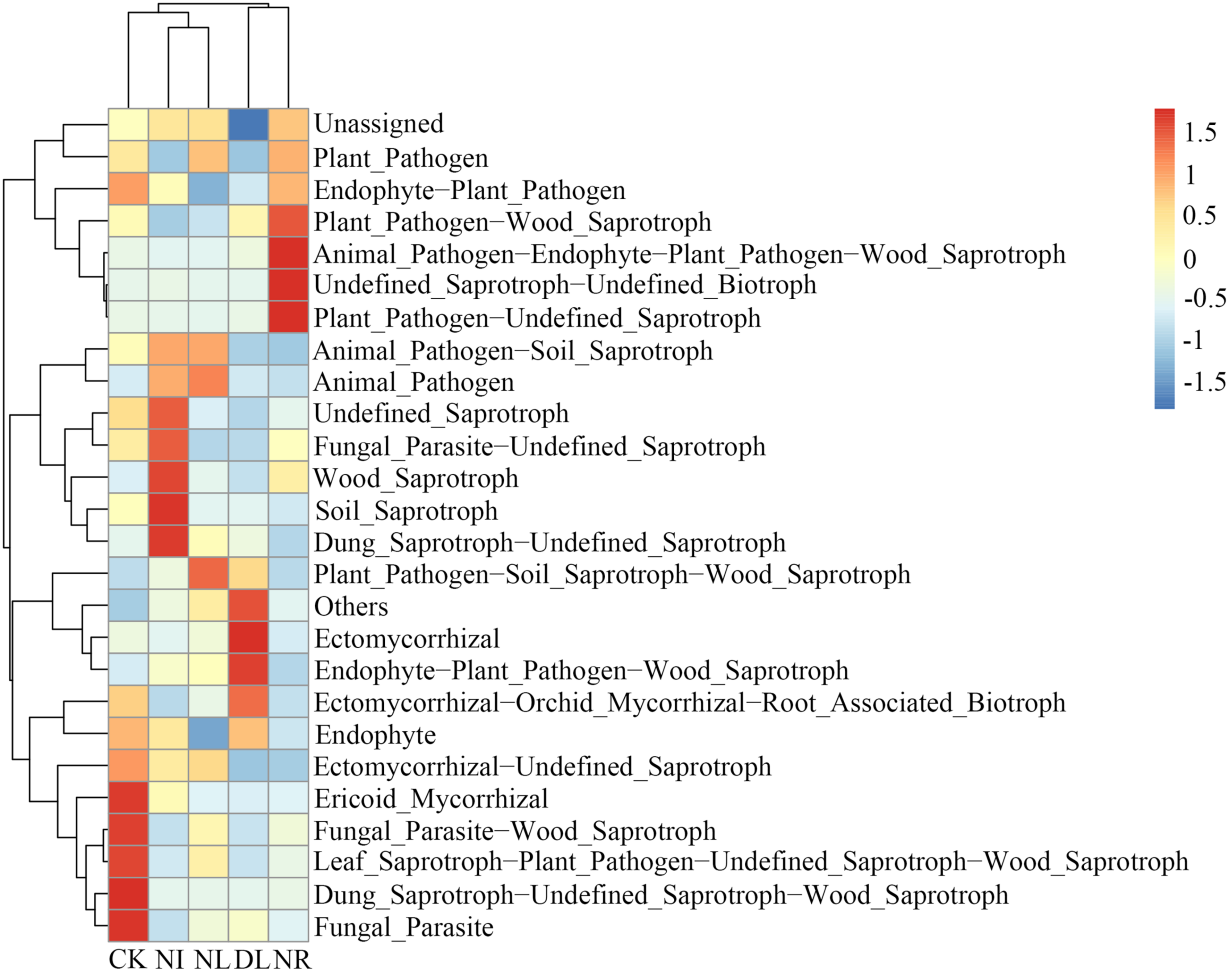
Changes in functional groups of soil fungi under different treatments. CK, DL, NL, NR, and NI represent control, doubled litter input, litter removal, root exclusion, root and litter exclusion, respectively.

For fungal communities, a total of 25 predicted functional guilds were detected under different treatments (Figure 7). The NI and NL treatments significantly decreased the relative abundance of ectomycorrhizal-orchid mycorrhizal-root associated biotrophs (NI: *p* = 0.01, NL: *p* = 0.02). The relative abundance of ectomycorrhizal (*p* = 0.04) and soil saprotrophs (*p* = 0.05) in the NR treatment was significantly lower than that in the CK.

### The Relationships Between Soil Physicochemical Factors and Soil Bacterial and Fungal Communities

**Figure 8:**
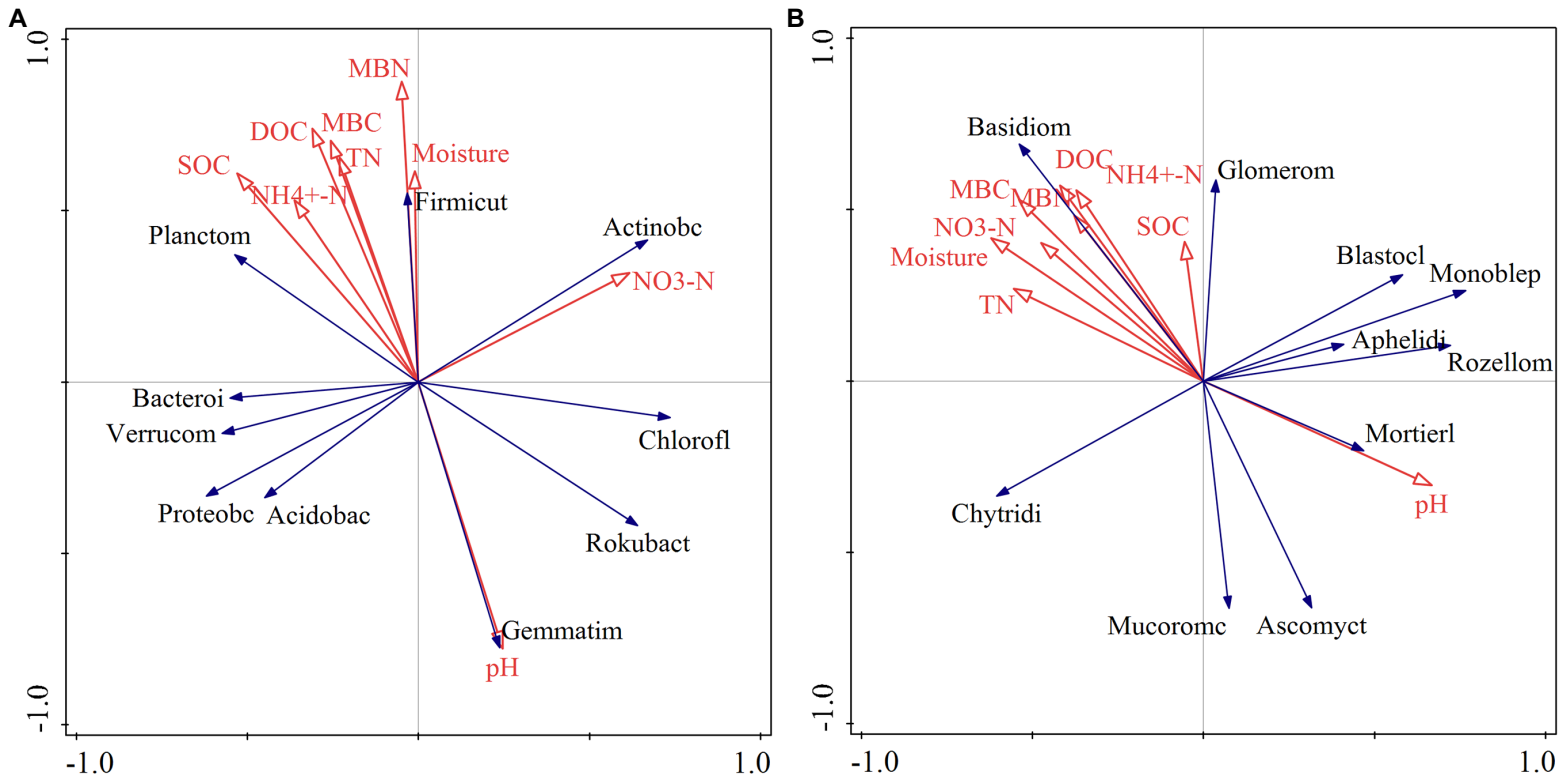
Redundancy analysis of soil bacterial **(A)** and fungal **(B)** communities and soil physicochemical properties. pH: soil pH; NO_3_^−^-N: soil nitrate nitrogen; moisture: soil moisture content; MBN: soil microbial biomass nitrogen; MBC: soil microbial biomass carbon; TN: total nitrogen; DOC: soil dissolved organic carbon; NH_4_^+^-N: soil ammonium nitrogen; SOC: soil organic carbon; Proteobc: Proteobacteria; Acidobac: Acidobacteria; Actinobacteria: Actinobacteria; Chlorofl: Chloroflexi; Bacteroi: Bacteroidetes; Verrucom: Verrucomicrobia; Planctom: Planctomycetes; Gemmatim: Gemmatimonadetes; Rokubact: Rokubacteria; Firmicut: Firmicutes; Basidiom: Basidiomycota; Ascomycot: Ascomycota; Mortierll: Mortierellomycota; Chytridi: Chytridiomycota; Rozellom: Rollomycota; Monhelep: Monoblephelomycota; Mucoromc: Mucoromycota; Blastocl: Blastocladiomycota; Glomerom: Glomeromycota; Aphelidi: Aphelidiomycota.

The RDA indicated that different relationships were detected between the dominant phyla in the bacterial community and soil physical and chemical factors (Figure 8). Soil Gemmatimonadetes was negatively correlated with soil C, N, and other nutrient elements and positively correlated with soil pH (Figure 8A). Soil dissolved organic carbon (DOC) and microbial biomass N showed a positive correlation with Firmicutes (Figure 8A). There was a positive correlation between soil nitrate N and Rokubacteria (Figure 8A). The Monte Carlo permutation test showed that soil organic C (*F* = 2.5, *p* = 0.038) was the main factor that drove changes in dominant phyla in the bacterial community (Table 1). Furthermore, the metabolism of cofactors and vitamins was significantly positively correlated with soil microbial biomass C (*p* < 0.05) and had a highly significantly positively correlated with ammonium N (*p* < 0.01; Supplementary Figure S2A).

**Table 1:** Explanatory quantity of soil physicochemical factor.

	Index	Interpretation of environmental factors/%	*F*	*p*
Soil bacteria	SOC	16.0	2.5	0.04
	NO_3_-N	15.3	2.3	0.04
	MBN	13.8	2.1	0.06
	DOC	13.7	2.1	0.05
	pH	12.7	1.9	0.07
	MBC	12.1	1.8	0.10
	NH_4_^+^-N	10.7	1.6	0.17
	TN	9.9	1.4	0.20
	Moisture	8.4	1.2	0.34
Soil fungi	pH	15.8	2.4	0.02
	Moisture	15.7	2.4	0.03
	MBC	15.5	2.4	0.02
	TN	14.2	2.1	0.04
	DOC	14	2.1	0.058
	NH_4_^+^-N	12.8	1.9	0.07
	NO_3_^−^-N	11.8	1.7	0.09
	MBN	10.9	1.6	0.12
	SOC	9.2	1.3	0.24

For fungal communities, Basidiomycota was positively correlated with microbial biomass C and total N (Figure 8B). Glomeromycota showed a positive relationship with soil microbial biomass N and ammonium N (Figure 8B). Soil water content exhibited a negative correlation with Monoblepharomycota. The effects of soil pH, water content, and microbial biomass C on the dominant phyla of soil fungi were greater than those of other factors (Table 1). For fungal function, soil ectomycorrhiza was significantly or highly significantly positively correlated with soil C, N and its fractions, but negatively correlated with soil pH (Supplementary Figure S2B).

## Discussion

### Effects of Litter and Root Manipulations on the Community Structure and Diversity of Soil Bacteria and Fungi

The structure of the soil microbial community was closely linked with changes in soil physicochemical factors (Ding et al., 2020). In this study, Proteobacteria, Acidobacteria, and Actinobacteria were the dominant soil bacteria in the different treatments (Figure 2A), which is consistent with the previous research results (Shang et al., 2021). Soil Proteobacteria, Acidobacteria, and Actinobacteria play an important role in the soil C and N cycles and organic matter decomposition, so these three classes of bacteria occupy a core status in the bacterial community (Eichorst et al., 2018; Song et al., 2021). Meanwhile, the soil bacterial community structure is also influenced by many factors, such as soil properties, litter, and roots (Yu et al., 2021). Our results showed that the relative abundance of Proteobacteria, Acidobacteria, and Planctomycetes in the NL and NR treatments was lower than that in the CK (Figure 2A). Previous study has shown that Proteobacteria are the major functional bacteria for the decomposition and transformation of organic matter (Aleinikoviene et al., 2017). NL and NR decreased the quantity of organic matter input into the soil and the availability of soil nutrients (Supplementary Table S2), thus leading to a decrease in the relative abundance of Proteobacteria (Liu et al., 2019). Soil Acidobacteria is well suited for survival in highly acidic environments (Mannisto et al., 2013). NL, NR, and NI blocked acidic substances such as phenolic acids and resins produced during the decomposition of roots and litter, resulting in an increase in soil pH (Dai et al., 2021). The loss of an acidic soil environment suppressed the colonization and growth of Acidobacteria and unidentified Acidobacteria. Generally, soil Actinobacteria can be enriched under drought conditions (Wipf et al., 2021). This study showed that NL and NR increased the relative abundance of Actinomycetes (Figure 2A). We attribute this finding to two mechanisms. First, the reduction in soil water content in the NL treatment caused Actinobacteria to alleviate drought stress by producing spores and filaments, which ultimately resulted in an increase in drought-tolerant Actinobacteria (Xiong et al., 2008). Second, soil Actinobacteria are associated with the degradation of refractory C, such as lignin, and thus Actinobacteria increased with the decrease of live roots (Griffiths et al., 1999). We also observed that the DL increased the relative abundance of soil Firmicutes (Figure 2A). This is because the addition of litter improved the soil nutrient status and hydrothermal environment and increased the availability of organic matter (Supplementary Table S2), which facilitates the involvement of Firmicutes in organic matter decomposition and carbohydrate metabolism (Zhao et al., 2017).

In this work, the dominant phyla of the soil fungal community under different treatments were Basidiomycota, Ascomycota, and Mortierellomycota (Figure 2B), which is consistent with the results of Tedersoo et al. (2014) on soil fungi in terrestrial ecosystems. Previous studies have indicated that Basidiomycota, Ascomycota, and Mortierellomycota, as important decomposers in soil, play an important role in the material cycle of forest soil and litter decomposition and show potent adaptive capacity (Baldrian, 2017; Zhang et al., 2018a). We also found that the relative abundance of soil Basidiomycota and Ramaria increased in the DL treatment compared with the CK (Figures 2B, 3). Basidiomycetes have the ability to degrade refractory organic matter such as lignin and are the main decomposers in lignin-rich forests, and Ramaria spp. is known to be an important taxa in Basidiomycota. The litter of Schrenk’s spruce forests contained a large amount of lignin, and the increase in lignin content under the DL treatment provided favorable conditions for the survival of Basidiomycetes and Ramaria (Klotzbucher et al., 2012). In addition, the relative abundance of Ascomycota decreased in the NL and NR treatments (Figure 2B). Ascomycetes play a prominent role in the decomposition of recalcitrant organic matter such as lignin and cutin, and their growth rate is closely related to the soil N content (Paungfoo-Lonhienne et al., 2015). The NL and NR treatments decreased the amount of recalcitrant organic matter and soil N content (Supplementary Table S2), thereby suppressing the growth and reproduction of Ascomycota (Li et al., 2021).

The change in soil microbial diversity is closely correlated with the stability of ecosystems and is influenced by multiple factors, such as litter, root exudates, and the soil environment (Sun et al., 2016). The bacterial Shannon index significantly decreased in the NI treatment (Figure 5), which is consistent with the previous findings (Zeng et al., 2017; Yang et al., 2020). Crow et al. (2009) suggested that changes in the quantity of litter and roots can affect soil microbial diversity by changing soil physicochemical properties. The removal of litter and roots hindered C input belowground, decreased soil microbial biomass C and the diversity of the C source available to the microorganisms, and consequently decreased the soil microbial alpha diversity index (Jing et al., 2021). Additionally, the removal of litter and roots reduce understory plant diversity and carbon source input by changing soil nutrients, thus inhibiting soil microbial diversity (Zhao et al., 2013). Another potential explanation for this reduction is the apoptosis of soil microbes caused by variation in soil hydrothermal conditions (Hamer et al., 2007). Soil is exposed to intense light and rain as litter and roots are removed (Kovacs et al., 2020), and microbial taxa with less tolerance to drought and high temperature in the soil can disappear. Drastic changes in soil hydrothermal factors may also damage the mycelium structure (Brant et al., 2006) and reduce the ability of soil microbes to utilize available, with a consequent reduction in microbial diversity (van der Heijden et al., 2015). Furthermore, the decrease in soil bacterial and fungal diversity and numbers under the DL treatment in this study could be attributed to the loss of specific microbial taxa caused by the decrease of soil pH (Figure 5). Previous studies shown that the reduction in soil pH under DL treatment increased the toxicity of aluminum and hydrogen ions in the soil and inhibited the ability of plants to release C into the soil (Chander and Brookes, 1991; Juhos et al., 2021). These changes reduced the transformation of plant C to microbial C and the growth of microbial taxa with less tolerance for acid (Pietri and Brookes, 2008).

### Effects of Litter and Root Manipulations on Functional Groups of Soil Bacteria and Fungi

Soil microbial functional groups can be indicative of environmental changes, providing a basis for a thorough understanding of microbe-mediated soil nutrient cycling (Chen et al., 2020). Metabolism groups maintain the growth of bacteria by acquiring energy, vitamins, and carbohydrates from the soil (Srour et al., 2020). In the present study, NI treatment decreased the expression of groups related to metabolic function in soil bacteria (e.g., metabolism of cofactors and vitamins; Figure 6). The NI treatment reduced not only the decomposition degree of plant detritus but also the content of humus and nutrient elements in the soil. The change in soil nutrients suppressed the metabolism of vitamins, enzymes and other substances, resulting in a decline in the abundance of functional groups (Wen et al., 2016). For lipid metabolism, the reduced abundance of lipid metabolism genes in NI treatment led to a decrease in fatty acid content (a marker of microbial membrane), which can weaken the ability of bacteria to cope with environmental stress (Srour et al., 2020). In addition, we observed that NL significantly reduced the functional groups associated with environmental adaptation, cell growth, and death (Figure 6). We speculate that removing litter reduced the input of aboveground organic matter and suppressed the growth of microorganisms related to the soil nutrient cycle (Sayer et al., 2020). Another potential explanation for our results is that litter removal leads to an increase in soil temperature and pH and a decrease in soil water content and nutrient content, eventually triggering changes in the environmental adaptability of bacteria (Gong et al., 2020; Dai et al., 2021).

In the fungal community, the relative abundance of ectomycorrhizae under DL treatment was significantly higher than that under CK (Figure 7). In general, ectomycorrhizae are symbiotic fungi, and their growth is susceptible to host plants. The increase in soil nutrients under DL treatment can promote growth of the belowground parts of plants, which in turn can provide a huge amount of organic matter for the metabolic activities of fungi (Liu et al., 2021). Moreover, DL treatment promotes good retention of soil moisture, and the appropriate soil environment can satisfy the conditions required for the growth of ectomycorrhizae (Zhu et al., 2021). Our present study also showed that NI and NL significantly reduced the relative abundance of ectomycorrhizal-orchid mycorrhizal root-associated biotrophs (Figure 7). This is mainly because the removal of litter and roots blocked the C source input into the soil and increased soil disturbance, and the survival conditions of fungi were stressed, which resulted in a decrease in the number of functional groups and the diversity of fungi of multiple trophic types (Lajtha et al., 2014; Liu et al., 2019). Furthermore, the relative abundances of ectomycorrhizal and soil saprotrophs under the NR treatment were lower than those under the CK treatment (Figure 7). The removal of litter and roots can decrease soil nutrient elements and accelerate soil water loss, and these changes can suppress the growth and metabolism of ectomycorrhizae (Wang et al., 2019). Previous studies have indicated that soil saprotrophs mainly maintain their growth by utilizing soil organic matter (Francioli et al., 2021). The removal of litter and roots reduced the amount of organic matter and the content of nutrients such as soil C and N, resulting in a decrease in soil saprotrophs.

### Relationship Between Soil Bacterial and Fungal Communities and Soil Physicochemical Factors

The change in the soil microbial community is closely related to soil nutrients and the environmental factors that affect microbial growth. In this study, there was a very significant negative correlation between Gemmatimonadetes and soil C, N, and other nutrient elements (Figure 8A). As Gram-negative bacteria, soil Gemmatimonadetes can degrade and consume C sources in soil and reduce the content of nutrient elements such as soil organic matter (Toda and Uchida, 2017). The increase in the relative abundance of soil Gemmatimonadetes under NL and NR treatments may have led to considerable consumption of organic matter, resulting in a decrease in soil nutrient content. Soil pH showed strong significant correlations with the relative abundance of Gemmatimonadetes in this study, consistent with results published by other authors (Liu et al., 2014). The availability of soil nutrients is the highest when the soil pH is neutral, which is beneficial for microbial growth. Although the soil pH changed (6.50–7.56) under different treatments, it remained in the suitable growth range of Gemmatimonadetes. In line with our initial hypothesis, soil organic C was the major driver of the change in the bacterial community, congruent with the previous findings (Chen et al., 2014). Litter and root manipulations altered the organic C content in the input soil and the energy source required for microbial activities, in turn affecting the bacterial community composition (Zhao et al., 2013).

For fungi, soil Basidiomycota showed a positive relationship with soil C and N (Figure 8B). It was reported that a large proportion of Basidiomycota in soil can form mycorrhizas with plant roots (Kumar and Atri, 2018). The existence of mycorrhizae can transfer the C source in the host plant to the soil on the one hand and promote the accumulation of C in the soil by protecting the C in the aggregate from erosion on the other (Clemmensen et al., 2013; Zhang et al., 2016). Moreover, the mycorrhizae formed by Basidiomycota can affect the soil’s inorganic N content by changing other microbial communities, and their dead hyphae can also release N to the soil (Veresoglou et al., 2012). Thus, soil Basidiomycota are closely related to soil C and N. In this study, Glomeromycota had a positive correlation with soil microbial biomass N and ammonium N (Figure 8B). The phylum Glomeromycota is an important symbiotic fungus of plants that can transport nutrient elements in soil to plants and promote the soil N uptake of trees (Sturmer and Kemmelmeier, 2021). This study also indicated that there was a negative correlation between soil water content and Monoblepharomycota (Figure 8B), which is related to the anaerobic characteristics of Monoblepharomycota. The removal of litter and roots increased the evaporation of soil moisture, and the decreased soil moisture improved the aeration conditions of the soil, thereby inhibiting the growth of the phylum Monoblepharomycota (Wu et al., 2019). Additionally, our results also indicated that soil pH and soil moisture had a greater effect on the soil fungal community than other factors. Soil pH affects the microbial utilization of nutrients and the soil environment by changing the form of compounds in the soil, thus acting on the fungal community structure (Ingwersen et al., 2008). Soil moisture is thought to be one of the vital substances influencing fungal growth. The changes in soil water affect the metabolism of fungal cells and their utilization of C and N resources by changing the flow of cell membranes and proteins (Zhang et al., 2014). Moreover, soil moisture can affect plant growth, oxygen content and soil gas diffusion, which results in differences in the growth and reproduction of different fungal taxa (Freeman et al., 2002).

Our study also showed that soil microbial biomass C and ammonium N were significantly positively correlated with metabolism of cofactors and vitamins (Supplementary Figure S2A). This follows because an increase in soil C, N, and other nutrients under DL treatment facilitated the metabolic process of cofactors and vitamins, terpenoids, and polyketides, and the enhancement of bacterial metabolic activity in turn had a positive effect on the accumulation of soil C (Ma et al., 2021). Conversely, the metabolic activity of bacteria was inhibited due to the blocking of soil C input under the NL and NR treatments (Supplementary Table S2). In this study, we observed that soil ectomycorrhiza was significantly or highly significantly positively correlated with soil C fractions (Supplementary Figure S2B). This was possibly because the following two aspects. Firstly, the turnover and biomass of ectomycorrhiza were important sources of soil C, and their mycorrhizal hyphae enhanced the physical protection of soil C by interacting with soil aggregates (Zhang et al., 2018b). Secondly, the secretion of ectomycorrhizal fungi promoted microbial activities and the decomposition of organic C, thus affecting the turnover of soil C (Hodge and Fitter, 2010). Similarly, we found that soil total N, ammonium N and microbial biomass N were significantly or highly significantly positively correlated with ectomycorrhizal-orchid mycorrhizal-root associated biotroph (Supplementary Figure S2B). We speculate that ectomycorrhizal fungi can degrade cellulose through a variety of enzyme genes and use their own oxidase to decompose organic matter in the soil, thereby releasing N into the soil (Shah et al., 2016).

## Conclusion

In this study, we analyzed the effects of 2 years of detritus input and removal treatment on the structure, diversity and function of the soil microbial community and explored the relationship between the microbial community and soil physicochemical properties. Our results indicate that the removal of litter and roots decreased the alpha diversity index of soil bacteria and fungi. The dominant phyla and functional groups in bacterial and fungal communities also exhibited some variances after different treatments. Our further analysis found that soil organic C, pH, and soil water content are important factors driving changes in bacterial and fungal communities in Schrenk’s spruce. Our study provides a basis for a deeper understanding of the role of soil microorganisms in the regulation of soil nutrient cycling in forest ecosystems. Future studies should focus on the relationship between microbial metabolic pathways and the soil nutrient cycle, which will help to improve the understanding of soil geochemical cycling mechanisms in forest ecosystems in the context of global change.

## Data Availability Statement

The data presented in the study are deposited in the National Center Biotechnology Information (NCBI) database repository, accession number PRJNA 814000.

## Author Contributions

HZ wrote the first draft of the manuscript. LG assisted with revising the draft manuscript. YL, JT, ZD, and XL carried out the field work. All authors contributed to the article and approved the submitted version.

## Funding

This research was funded by the Key Laboratory Open Topic of Xinjiang Uygur Autonomous Region (no. 2019D04001), the National Natural Science Foundation of China (no. 31760142), and Xinjiang Uygur Autonomous Region Graduate Research and Innovation Project (no. XJ2020G010).

## Conflict of Interest

The authors declare that the research was conducted in the absence of any commercial or financial relationships that could be construed as a potential conflict of interest.

## Publisher’s Note

All claims expressed in this article are solely those of the authors and do not necessarily represent those of their affiliated organizations, or those of the publisher, the editors and the reviewers. Any product that may be evaluated in this article, or claim that may be made by its manufacturer, is not guaranteed or endorsed by the publisher.

## Footnotes

^1^ http://www.drive5.com/uparse/

## References

[ref1] Aleinikoviene J. Armolaitis K. Cesnuleviciene R. Zekaite V. Muraskiene M. (2017). The status of soil organic matter decomposing microbiota in afforested and abandoned arable Arenosols. Zemdirbyste-Agriculture 104, 195–202. doi: 10.13080/z-a.2017.104.025

[ref2] Allison S. D. Martiny J. B. H. (2008). Resistance, resilience, and redundancy in microbial communities. Proc. Natl. Acad. Sci. U. S. A. 105, 11512–11519. doi: 10.1073/pnas.0801925105, PMID: 18695234 PMC2556421

[ref3] Baldrian P. (2017). Forest microbiome: diversity, complexity and dynamics. FEMS Microbiol. Rev. 41, 109–130. doi: 10.1093/femsre/fuw040, PMID: 27856492

[ref4] Barberan A. McGuire K. L. Wolf J. A. Jones F. A. Wright S. J. Turner B. L.. (2015). Relating belowground microbial composition to the taxonomic, phylogenetic, and functional trait distributions of trees in a tropical forest. Ecol. Lett. 18, 1397–1405. doi: 10.1111/ele.12536, PMID: 26472095

[ref5] Bardgett R. D. van der Putten W. H. (2014). Belowground biodiversity and ecosystem functioning. Nature 515, 505–511. doi: 10.1038/nature13855 25428498

[ref6] Brant J. B. Sulzman E. W. Myrold D. D. (2006). Microbial community utilization of added carbon substrates in response to long-term carbon input manipulation. Soil Biol. Biochem. 38, 2219–2232. doi: 10.1016/j.soilbio.2006.01.022

[ref7] China soil systematic classification research cooperation group (1995). China Soil System Classifcation (Amendment Scheme). Beijing: Agricultural Science And Technology Press Of China.

[ref8] Cantarel A. A. M. Pommier T. Desclos-Theveniau M. Diquelou S. Dumont M. Grassein F.. (2015). Using plant traits to explain plant-microbe relationships involved in nitrogen acquisition. Ecology 96, 788–799. doi: 10.1890/13-2107.1, PMID: 26236874

[ref9] Caporaso J. G. Kuczynski J. Stombaugh J. Bittinger K. Bushman F. D. Costello E. K.. (2010). QIIME allows analysis of high-throughput community sequencing data. Nat. Methods 7, 335–336. doi: 10.1038/nmeth.f.303, PMID: 20383131 PMC3156573

[ref10] Chander K. Brookes P. C. (1991). Plant inputs of carbon to metal-contaminated soil and effects on the soil microbial biomass. Soil Biol. Biochem. 23, 1169–1177. doi: 10.1016/0038-0717(91)90030-N

[ref11] Che R. X. Liu D. Qin J. L. Wang F. Wang W. J. Xu Z. H.. (2020). Increased litter input significantly changed the total and active microbial communities in degraded grassland soils. J. Soils Sediments 20, 2804–2816. doi: 10.1007/s11368-020-02619-x

[ref12] Chen Q. L. Ding J. Li C. Y. Yan Z. Z. He J. Z. Hu H. W. (2020). Microbial functional attributes, rather than taxonomic attributes, drive top soil respiration, nitrification and denitrification processes. Sci. Total Environ. 734, 139479. doi: 10.1016/j.scitotenv.2020.139479, PMID: 32464393

[ref13] Chen X. Gong L. Liu Y. T. (2018). The ecological stoichiometry and interrelationship between litter and soil under seasonal snowfall in Tianshan Mountain. Ecosphere 9, e02520. doi: 10.1002/ecs2.2520

[ref14] Chen L. Zhang J. B. Zhao B. Z. Yan P. Zhou G. X. Xin X. L. (2014). Effects of straw amendment and moisture on microbial communities in Chinese fluvo-aquic soil. J. Soils Sediments 14, 1829–1840. doi: 10.1007/s11368-014-0924-2

[ref15] Clemmensen K. E. Bahr A. Ovaskainen O. Dahlberg A. Ekblad A. Wallander H.. (2013). Roots and associated fungi drive long-term carbon sequestration in boreal forest. Science 339, 1615–1618. doi: 10.1126/science.1231923, PMID: 23539604

[ref16] Cowan P. J. (2007). Geographic usage of the terms middle Asia and Central Asia. J. Arid Environ. 69, 359–363. doi: 10.1016/j.jaridenv.2006.09.013

[ref17] Crow S. E. Lajtha K. Bowden R. D. Yano Y. Brant J. B. Caldwell B. A.. (2009). Increased coniferous needle inputs accelerate decomposition of soil carbon in an old-growth forest. For. Ecol. Manag. 258, 2224–2232. doi: 10.1016/j.foreco.2009.01.014

[ref18] Crowther T. W. Sokol N. W. Oldfield E. E. Maynard D. S. Thomas S. M. Bradford M. A. (2015). Environmental stress response limits microbial necromass contributions to soil organic carbon. Soil Biol. Biochem. 85, 153–161. doi: 10.1016/j.soilbio.2015.03.002

[ref19] Dai W. Peng B. Liu J. Wang C. Wang X. Jiang P.. (2021). Four years of litter input manipulation changes soil microbial characteristics in a temperate mixed forest. Biogeochemistry 154, 371–383. doi: 10.1007/s10533-021-00792-w

[ref20] Dilly O. Bloem J. Vos A. Munch J. C. (2004). Bacterial diversity in agricultural soils during litter decomposition. Appl. Environ. Microbiol. 70, 468–474. doi: 10.1128/aem.70.1.468-474.2004, PMID: 14711676 PMC321295

[ref21] Ding L. L. Shang Y. S. Zhang W. Zhang Y. Li S. G. Wei X.. (2020). Disentangling the effects of driving forces on soil bacterial and fungal communities under shrub encroachment on the Guizhou plateau of China. Sci. Total Environ. 709:136207. doi: 10.1016/j.scitotenv.2019.136207, PMID: 31887509

[ref22] Eichorst S. A. Trojan D. Roux S. Herbold C. Rattei T. Woebken D. (2018). Genomic insights into the Acidobacteria reveal strategies for their success in terrestrial environments. Environ. Microbiol. 20, 1041–1063. doi: 10.1111/1462-2920.14043, PMID: 29327410 PMC5900883

[ref23] Fanin N. Bertrand I. (2016). Aboveground litter quality is a better predictor than belowground microbial communities when estimating carbon mineralization along a land-use gradient. Soil Biol. Biochem. 94, 48–60. doi: 10.1016/j.soilbio.2015.11.007

[ref24] Feng J. Li Z. Hao Y. Wang J. Ru J. Song J.. (2022). Litter removal exerts greater effects on soil microbial community than understory removal in a subtropical-warm temperate climate transitional forest. For. Ecol. Manag. 505:119867. doi: 10.1016/j.foreco.2021.119867

[ref25] Francioli D. van Rijssel S. Q. van Ruijven J. Termorshuizen A. J. Cotton T. E. A. Dumbrell A. J.. (2021). Plant functional group drives the community structure of saprophytic fungi in a grassland biodiversity experiment. Plant Soil 461, 91–105. doi: 10.1007/s11104-020-04454-y

[ref26] Freeman C. Nevison G. B. Kang H. Hughes S. Reynolds B. Hudson J. A. (2002). Contrasted effects of simulated drought on the production and oxidation of methane in a mid-Wales wetland. Soil Biol. Biochem. 34, 61–67. doi: 10.1016/s0038-0717(01)00154-7

[ref27] Gong C. Song C. C. Sun L. Zhang D. Zhang J. Liu X. H. (2020). Response of methane emissions to litter input manipulation in a temperate freshwater marsh, Northeast China. Ecol. Indic. 115, 106377. doi: 10.1016/j.ecolind.2020.106377

[ref28] Gong L. Zhao J. J. (2019). The response of fine root morphological and physiological traits to added nitrogen in Schrenk’s spruce (Picea schrenkiana) of the Tianshan mountains, China. PeerJ 7, 20. doi: 10.7717/peerj.8194, PMID: 31824779 PMC6898987

[ref29] Griffiths B. Ritz K. Ebblewhite N. Dobson G. (1999). Soil microbial community structure: effects of substrate loading rates. Soil Biol. Biochem. 31, 145–153. doi: 10.1016/S0038-0717(98)00117-5

[ref30] Habtewold J. Z. Helgason B. L. Yanni S. F. Janzen H. H. Ellert B. H. Gregorich E. G. (2020). Litter composition has stronger influence on the structure of soil fungal than bacterial communities. Eur. J. Soil Biol. 98:103190. doi: 10.1016/j.ejsobi.2020.103190

[ref31] Hamer U. Unger M. Makeschin F. (2007). Impact of air-drying and rewetting on PLFA profiles of soil microbial communities. J. Plant Nutr. Soil Sci. 170, 259–264. doi: 10.1002/jpln.200625001

[ref32] Han S. Delgado-Baquerizo M. Luo X. S. Liu Y. R. Van Nostrand J. D. Chen W. L.. (2021). Soil aggregate size-dependent relationships between microbial functional diversity and multifunctionality. Soil Biol. Biochem. 154:108143. doi: 10.1016/j.soilbio.2021.108143

[ref33] Hodge A. Fitter A. H. (2010). Substantial nitrogen acquisition by arbuscular mycorrhizal fungi from organic material has implications for N cycling. Proc. Natl. Acad. Sci. U. S. A. 107, 13754–13759. doi: 10.1073/pnas.1005874107, PMID: 20631302 PMC2922220

[ref34] Ingwersen J. Poll C. Streck T. Kandeler E. (2008). Micro-scale modelling of carbon turnover driven by microbial succession at a biogeochemical interface. Soil Biol. Biochem. 40, 864–878. doi: 10.1016/j.soilbio.2007.10.018

[ref35] Jing Y. L. Tian P. Wang Q. K. Li W. B. Sun Z. L. Yang H. (2021). Effects of root dominate over aboveground litter on soil microbial biomass in global forest ecosystems. For. Ecosyst. 8, 9. doi: 10.1186/s40663-021-00318-8

[ref36] Juhos K. Madarasz B. Kotroczo Z. Beni A. Makadi M. Fekete I. (2021). Carbon sequestration of forest soils is reflected by changes in physicochemical soil indicators - A comprehensive discussion of a long-term experiment on a detritus manipulation. Geoderma 385, 114918. doi: 10.1016/j.geoderma.2020.114918

[ref37] Klotzbucher T. Kaiser K. Stepper C. van Loon E. Gerstberger P. Kalbitz K. (2012). Long-term litter input manipulation effects on production and properties of dissolved organic matter in the forest floor of a Norway spruce stand. Plant Soil 355, 407–416. doi: 10.1007/s11104-011-1123-1

[ref38] Kovacs B. Tinya F. Nemeth C. Odor P. (2020). Unfolding the effects of different forestry treatments on microclimate in oak forests: results of a 4-yr experiment. Ecol. Appl. 30, e02043. doi: 10.1002/eap.2043, PMID: 31758609 PMC7900960

[ref39] Kumar J. Atri N. S. (2018). Studies on Ectomycorrhiza: An appraisal. Bot. Rev. 84, 108–155. doi: 10.1007/s12229-017-9196-z

[ref40] Lajtha K. Townsend K. L. Kramer M. G. Swanston C. Bowden R. D. Nadelhoffer K. (2014). Changes to particulate versus mineral-associated soil carbon after 50 years of litter manipulation in forest and prairie experimental ecosystems. Biogeochemistry 119, 341–360. doi: 10.1007/s10533-014-9970-5

[ref41] Langille M. G. I. Zaneveld J. Caporaso J. G. McDonald D. Knights D. Reyes J. A.. (2013). Predictive functional profiling of microbial communities using 16S rRNA marker gene sequences. Nat. Biotechnol. 31, 814–821. doi: 10.1038/nbt.2676, PMID: 23975157 PMC3819121

[ref42] Li Y. Liu X. J. Xu W. B. Bongers F. J. Bao W. K. Chen B.. (2020). Effects of diversity, climate and litter on soil organic carbon storage in subtropical forests. For. Ecol. Manag. 476, 118479. doi: 10.1016/j.foreco.2020.118479

[ref43] Li S. Song M. Jing S. (2021). Effects of different carbon inputs on soil nematode abundance and community composition. Appl. Soil Ecol. 163, 103915. doi: 10.1016/j.apsoil.2021.103915

[ref44] Liu X. Lin T.-C. Vadeboncoeur M. A. Yang Z. Chen S. Xiong D.. (2019). Root litter inputs exert greater influence over soil C than does aboveground litter in a subtropical natural forest. Plant Soil 444, 489–499. doi: 10.1007/s11104-019-04294-5

[ref45] Liu M. Liu J. Jiang C. Y. Wu M. Song R. S. Gui R. Y.. (2017). Improved nutrient status affects soil microbial biomass, respiration, and functional diversity in a lei bamboo plantation under intensive management. J. Soils Sediments 17, 917–926. doi: 10.1007/s11368-016-1603-2

[ref46] Liu J. J. Sui Y. Y. Yu Z. H. Shi Y. Chu H. Y. Jin J.. (2014). High throughput sequencing analysis of biogeographical distribution of bacterial communities in the black soils of Northeast China. Soil Biol. Biochem. 70, 113–122. doi: 10.1016/j.soilbio.2013.12.014

[ref47] Liu R. Zhang Y. Hu X. F. Wan S. Z. Wang H. M. Liang C.. (2021). Litter manipulation effects on microbial communities and enzymatic activities vary with soil depth in a subtropical Chinese fir plantation. For. Ecol. Manag. 480:118641. doi: 10.1016/j.foreco.2020.118641

[ref48] Lu L. H. Yin S. X. Liu X. Zhang W. M. Gu T. Y. Shen Q. R.. (2013). Fungal networks in yield-invigorating and -debilitating soils induced by prolonged potato monoculture. Soil Biol. Biochem. 65, 186–194. doi: 10.1016/j.soilbio.2013.05.025

[ref49] Ma X. Luo Z. Z. Zhang Y. Q. Liu J. H. Niu Y. N. Cai L. Q. (2021). Distribution characteristics and ecological function predictions of soil bacterial communities in rainfed alfalfa fields on the loess plateau. Acta Pratacul. Sin. 30, 54–67. doi: 10.11686/cyxb2020381

[ref50] Maillard F. Leduc V. Bach C. Reichard A. Fauchery L. Saint-Andre L.. (2019). Soil microbial functions are affected by organic matter removal in temperate deciduous forest. Soil Biol. Biochem. 133, 28–36. doi: 10.1016/j.soilbio.2019.02.015

[ref51] Mannisto M. K. Kurhela E. Tiirola M. Haggblom M. M. (2013). Acidobacteria dominate the active bacterial communities of Arctic tundra with widely divergent winter-time snow accumulation and soil temperatures. FEMS Microbiol. Ecol. 84, 47–59. doi: 10.1111/1574-6941.12035, PMID: 23106413

[ref52] Nguyen N. H. Song Z. Bates S. T. Branco S. Tedersoo L. Menke J.. (2016). FUNGuild: An open annotation tool for parsing fungal community datasets by ecological guild. Fungal Ecol. 20, 241–248. doi: 10.1016/j.funeco.2015.06.006

[ref53] Paungfoo-Lonhienne C. Yeoh Y. K. Kasinadhuni N. R. P. Lonhienne T. G. A. Robinson N. Hugenholtz P.. (2015). Nitrogen fertilizer dose alters fungal communities in sugarcane soil and rhizosphere. Sci. Rep. 5, 6. doi: 10.1038/srep08678, PMID: 25728892 PMC5155403

[ref54] Peiffer J. A. Spor A. Koren O. Jin Z. Tringe S. G. Dangl J. L.. (2013). Diversity and heritability of the maize rhizosphere microbiome under field conditions. Proc. Natl. Acad. Sci. U. S. A. 110, 6548–6553. doi: 10.1073/pnas.1302837110, PMID: 23576752 PMC3631645

[ref55] Pietri J. C. A. Brookes P. C. (2008). Relationships between soil pH and microbial properties in a UK arable soil. Soil Biol. Biochem. 40, 1856–1861. doi: 10.1016/j.soilbio.2008.03.020

[ref56] Pisani O. Lin L. H. Lun O. O. Y. Lajtha K. Nadelhoffer K. J. Simpson A. J.. (2016). Long-term doubling of litter inputs accelerates soil organic matter degradation and reduces soil carbon stocks. Biogeochemistry 127, 1–14. doi: 10.1007/s10533-015-0171-7

[ref57] Santiago T. Pablo P. Olga C. Veronica G. Marina G. P. (2021). Soil microbial communities respond to an environmental gradient of grazing intensity in South Patagonia Argentina. J. Arid Environ. 184:104300. doi: 10.1016/j.jaridenv.2020.104300

[ref58] Sauvadet M. Fanin N. Chauvat M. Bertrand I. (2019). Can the comparison of above-and below-ground litter decomposition improve our understanding of bacterial and fungal successions? Soil biol. Biochemist 132, 24–27. doi: 10.1016/j.soilbio.2019.01.022

[ref59] Sayer E. J. Rodtassana C. Sheldrake M. Brechet L. M. Ashford O. S. Lopez-Sangil L.. (2020). “Revisiting nutrient cycling by litterfall-insights from 15 years of litter manipulation in old-growth lowland tropical forest,” in Tropical Ecosystems in the 21st Century. eds. Dumbrell A. J. Turner E. C. Fayle T. M., 173–223.

[ref60] Scarlett K. Denman S. Clark D. R. Forster J. Vanguelova E. Brown N.. (2021). Relationships between nitrogen cycling microbial community abundance and composition reveal the indirect effect of soil pH on oak decline. ISME J. 15, 623–635. doi: 10.1038/s41396-020-00801-0, PMID: 33067585 PMC8027100

[ref61] Schroeder J. Jannoura R. Beuschel R. Pfeiffer B. Dyckmans J. Murugan R.. (2020). Carbon use efficiency and microbial functional diversity in a temperate Luvisol and a tropical Nitisol after millet litter and N addition. Biol. Fertil. Soils 56, 1139–1150. doi: 10.1007/s00374-020-01487-4

[ref62] Shah F. Nicolas C. Bentzer J. Ellstrom M. Smits M. Rineau F.. (2016). Ectomycorrhizal fungi decompose soil organic matter using oxidative mechanisms adapted from saprotrophic ancestors. New Phytol. 209, 1705–1719. doi: 10.1111/nph.13722, PMID: 26527297 PMC5061094

[ref63] Shang R. Li S. Huang X. Liu W. Lang X. Su J. (2021). Effects of soil properties and plant diversity on soil microbial community composition and diversity during secondary succession. Forests 12. doi: 10.3390/f12060805

[ref64] Shi S. J. Herman D. J. He Z. L. Pett-Ridge J. Wu L. Y. Zhou J. Z.. (2018). Plant roots alter microbial functional genes supporting root litter decomposition. Soil Biol. Biochem. 127, 90–99. doi: 10.1016/j.soilbio.2018.09.013

[ref65] Song D. D. Ren L. Li X. Ma D. L. Zang S. Y. (2021). Soil bacterial diversity and composition of different forest types in greater Xing’an mountains, China. Appl. Ecol. Environ. Res. 19, 1983–1997. doi: 10.15666/aeer/1903_19831997

[ref66] Srour A. Y. Ammar H. A. Subedi A. Pimentel M. Cook R. L. Bond J.. (2020). Microbial communities associated With Long-term tillage and fertility treatments in a corn-soybean cropping system. Front. Microbiol. 11:1363. doi: 10.3389/fmicb.2020.01363, PMID: 32670235 PMC7330075

[ref67] Storch D. Bohdalkova E. Okie J. (2018). The more-individuals hypothesis revisited: the role of community abundance in species richness regulation and the productivity-diversity relationship. Ecol. Lett. 21, 920–937. doi: 10.1111/ele.12941, PMID: 29659144

[ref68] Sturmer S. L. Kemmelmeier K. (2021). The Glomeromycota in the Neotropics. Front. Microbiol. 11:553679. doi: 10.3389/fmicb.2020.553679, PMID: 33510711 PMC7835493

[ref69] Sun L. Lu Y. Kronzucker H. J. Shi W. (2016). Quantification and enzyme targets of fatty acid amides from duckweed root exudates involved in the stimulation of denitrification. J. Plant Physiol. 198, 81–88. doi: 10.1016/j.jplph.2016.04.010, PMID: 27152459

[ref70] Tedersoo L. Bahram M. Polme S. Koljalg U. Yorou N. S. Wijesundera R., et al. (2014). Global diversity and geography of soil fungi. Science 346, 1078. doi: 10.1126/science.1256688 25430773

[ref71] Toda M. Uchida Y. (2017). Long-term use of green manure legume and chemical fertiliser affect soil bacterial community structures but not the rate of soil nitrate decrease when excess carbon and nitrogen are applied. Soil Res. 55, 524–533. doi: 10.1071/sr17109

[ref72] van der Heijden M. G. A. Martin F. M. Selosse M. A. Sanders I. R. (2015). Mycorrhizal ecology and evolution: the past, the present, and the future. New Phytol. 205, 1406–1423. doi: 10.1111/nph.13288, PMID: 25639293

[ref73] Veen G. F. ten Hooven F. C. Weser C. Hannula S. E. (2021). Steering the soil microbiome by repeated litter addition. J. Ecol. 109, 2499–2513. doi: 10.1111/1365-2745.13662

[ref74] Veresoglou S. D. Chen B. D. Rillig M. C. (2012). Arbuscular mycorrhiza and soil nitrogen cycling. Soil Biol. Biochem. 46, 53–62. doi: 10.1016/j.soilbio.2011.11.018

[ref75] Wan X. H. Chen X. L. Huang Z. Q. Chen H. Y. H. (2021). Contribution of root traits to variations in soil microbial biomass and community composition. Plant Soil 460, 483–495. doi: 10.1007/s11104-020-04788-7

[ref76] Wang Q. He T. Wang S. Liu L. (2013). Carbon input manipulation affects soil respiration and microbial community composition in a subtropical coniferous forest. Agric. For. Meteorol. 178-179, 152–160. doi: 10.1016/j.agrformet.2013.04.021

[ref77] Wang L. X. Pang X. Y. Li N. Qi K. B. Huang J. S. Yin C. Y. (2020a). Effects of vegetation type, fine and coarse roots on soil microbial communities and enzyme activities in eastern Tibetan plateau. Catena 194, 104694. doi: 10.1016/j.catena.2020.104694

[ref78] Wang Y. Zhang C. Zhang G. N. Wang L. Z. Gao Y. Wang X. L.. (2019). Carbon input manipulations affecting microbial carbon metabolism in temperate forest soils—A comparative study between broadleaf and coniferous plantations. Geoderma 355, 113914. doi: 10.1016/j.geoderma.2019.113914

[ref79] Wang Y. Zheng H. Yang Y. F. Liang Y. T. Zhou J. Z. He Z. L.. (2020b). Microbial functional gene diversity in natural secondary forest Ultisols. Acta Oecol.-Int. J. Ecol. 105, 103575. doi: 10.1016/j.actao.2020.103575

[ref80] Wen X. Y. Dubinsky E. Wu Y. Yu R. Chen F. (2016). Wheat, maize and sunflower cropping systems selectively influence bacteria community structure and diversity in their and succeeding crop’s rhizosphere. J. Integr. Agric. 15, 1892–1902. doi: 10.1016/s2095-3119(15)61147-9

[ref81] Wipf H. M. L. Thao-Nguyen B. Coleman-Derr D. (2021). Distinguishing Between the impacts of heat and drought stress on the root microbiome of Sorghum bicolor. Phytobiomes J. 5, 166–176. doi: 10.1094/pbiomes-07-20-0052-r

[ref82] Wirthner S. Frey B. Busse M. D. Schuetz M. Risch A. C. (2011). Effects of wild boar (*Sus scrofa* L.) rooting on the bacterial community structure in mixed-hardwood forest soils in Switzerland. Eur. J. Soil Biol. 47, 296–302. doi: 10.1016/j.ejsobi.2011.07.003

[ref83] Wu J. J. Lu M. Fenga J. Zhang D. D. Chen Q. Li Q. X.. (2019). Soil net methane uptake rates in response to short-term litter input change in a coniferous forest ecosystem of Central China. Agric. For. Meteorol. 271, 307–315. doi: 10.1016/j.agrformet.2019.03.017

[ref84] Xiong Y. Xia H. Li Z. A. Cai X. A. Fu S. (2008). Impacts of litter and understory removal on soil properties in a subtropical Acacia mangium plantation in China. Plant Soil 304, 179–188. doi: 10.1007/s11104-007-9536-6

[ref85] Xu S. Liu L. L. Sayer E. J. (2013). Variability of above-ground litter inputs alters soil physicochemical and biological processes: a meta-analysis of litterfall-manipulation experiments. BGeo 10, 7423–7433. doi: 10.5194/bg-10-7423-2013

[ref86] Xu M. Lu X. Xu Y. Zhong Z. Zhang W. Ren C.. (2020). Dynamics of bacterial community in litter and soil along a chronosequence of Robinia pseudoacacia plantations. Sci. Total Environ. 703, 135613. doi: 10.1016/j.scitotenv.2019.135613, PMID: 31761359

[ref87] Xu H. D. Yu M. K. Cheng X. R. (2021). Abundant fungal and rare bacterial taxa jointly reveal soil nutrient cycling and multifunctionality in uneven-aged mixed plantations. Ecol. Indic. 129, 107932. doi: 10.1016/j.ecolind.2021.107932

[ref88] Yan B. S. Sun L. P. Li J. J. Liang C. Q. Wei F. R. Xue S.. (2020). Change in composition and potential functional genes of soil bacterial and fungal communities with secondary succession in Quercus liaotwigensis forests of the Loess Plateau, western China. Geoderma 364, 114199. doi: 10.1016/j.geoderma.2020.114199

[ref89] Yang L. Wang N. Chen Y. Yang W. Tian D. S. Zhang C. Y.. (2020). Carbon management practices regulate soil bacterial communities in response to nitrogen addition in a pine forest. Plant Soil 452, 137–151. doi: 10.1007/s11104-020-04570-9

[ref90] Yu H. Zhang L. Wang Y. Xu S. Liu Y. Wang S. (2021). Response of soil bacterial communities to organic carbon input under soil freeze-thaw in forest ecosystems. Eur. J. Soil Biol. 105, 103333. doi: 10.1016/j.ejsobi.2021.103333

[ref91] Zeng Q. C. Liu Y. An S. S. (2017). Impact of litter quantity on the soil bacteria community during the decomposition of Quercus wutaishanica litter. PeerJ 5, 18. doi: 10.7717/peerj.3777, PMID: 28894648 PMC5592084

[ref92] Zhang B. W. Li S. Chen S. P. Ren T. T. Yang Z. Q. Zhao H. L.. (2016). Arbuscular mycorrhizal fungi regulate soil respiration and its response to precipitation change in a semiarid steppe. Sci. Rep. 6:19990. doi: 10.1038/srep19990, PMID: 26818214 PMC4730203

[ref93] Zhang N. L. Li Y. N. Wubet T. Bruelheide H. Liang Y. Purahong W.. (2018a). Tree species richness and fungi in freshly fallen leaf litter: unique patterns of fungal species composition and their implications for enzymatic decomposition. Soil Biol. Biochem. 127, 120–126. doi: 10.1016/j.soilbio.2018.09.023

[ref94] Zhang Z. Xiao J. Yuan Y. Zhao C. Liu Q. Yin H. (2018b). Mycelium- and root-derived C inputs differ in their impacts on soil organic C pools and decomposition in forests. Soil Biol. Biochem. 123, 257–265. doi: 10.1016/j.soilbio.2018.05.015

[ref95] Zhang X. F. Xu S. J. Li C. M. Zhao L. Feng H. Y. Yue G. Y.. (2014). The soil carbon/nitrogen ratio and moisture affect microbial community structures in alkaline permafrost-affected soils with different vegetation types on the Tibetan plateau. Res. Microbiol. 165, 128–139. doi: 10.1016/j.resmic.2014.01.002, PMID: 24463013

[ref96] Zhao Q. Classen A. T. Wang W. W. Zhao X. R. Mao B. Zeng D. H. (2017). Asymmetric effects of litter removal and litter addition on the structure and function of soil microbial communities in a managed pine forest. Plant Soil 414, 81–93. doi: 10.1007/s11104-016-3115-7

[ref97] Zhao J. Wan S. Z. Fu S. L. Wang X. L. Wang M. Liang C. F.. (2013). Effects of understory removal and nitrogen fertilization on soil microbial communities in eucalyptus plantations. For. Ecol. Manag. 310, 80–86. doi: 10.1016/j.foreco.2013.08.013

[ref98] Zhou X. Sun H. Sietio O. M. Pumpanen J. Heinonsalo J. Koster K.. (2020). Wildfire effects on soil bacterial community and its potential functions in a permafrost region of Canada. Appl. Soil Ecol. 156, 103713. doi: 10.1016/j.apsoil.2020.103713

[ref99] Zhu H. Q. Gong L. Ding Z. L. Li Y. F. (2021). Effects of litter and root manipulations on soil carbon and nitrogen in a Schrenk’s spruce (*Picea schrenkiana*) forest. PLoS One 16:e0247725. doi: 10.1371/journal.pone.0247725, PMID: 33630965 PMC7906406

