# Supplementary material for: Effects of Litter and Root Manipulations on Soil Bacterial and Fungal Community Structure and Function in a Schrenk’s Spruce (Picea schrenkiana) Forest
Source: Front Plant Sci. 2022 Apr 14;13:849483. doi: 10.3389/fpls.2022.849483 (PMC9047989; doi:10.3389/fpls.2022.849483)
Supplement: Supplementary file 1 [file Data_Sheet_1.ZIP › experimental data/Figure S1,2.doc]

**Figure S1.** Good’s coverage of soil bacteria (**A**) and fungi (**B**) under different treatments (n = 3). CK, DL, NL, NR and NI represent control, doubled litter

input, litter removal, root exclusion, root and litter exclusion, respectively.

**Figure S2.** The relationship between soil physicochemical properties and bacterial (A) and fungal (B) functional groups. ^***^ *p* <0.001, ^**^ 0.001<*p*<0.01, ^*^ 0.01<p<0.05

Note: ENS: Endocrine_System, AAM: Amino_Acid_Metabolism, MCV: Metabolism_of_Cofactors_and_Vitamins, CIP: Genetic_Information_Processing, PC: Poorly_Characterized, CAM: Carbohydrate_Metabolism; EM: Energy_Metabolism, CM: Cell_Motility, MT: Membrane_Transport, TR: Translation, EA: Environmental_Adaptation, MOAA: Metabolism_of_Other_Amino_Acids, SMI: Signaling_Molecules_and_Interaction, EXS: Excretory_System, SIT: Signal_Transduction, FSD: Folding,_Sorting_and_Degradation , LIM: Lipid_Metabolism, NED: Neurodegenerative_Diseases, BOSM: Biosynthesis_of_Other_Secondary_Metabolites, TRA: Transcription, MET: Metabolism, CAN: Cancers, TRC: Transport_and_Catabolism, NS: Nervous_System, XBM: Xenobiotics_Biodegradation_and_Metabolism, MD: Metabolic_Diseases, EF: Enzyme_Families, ISD: Immune_System_Diseases, CPS: Cellular_Processes_and_Signaling, MTP: Metabolism_of_Terpenoids_and_Polyketides, RR: Replication_and_Repair, NM: Nucleotide_Metabolism, CGD: Cell_Growth_and_Death, IND: Infectious_Diseases, GBM: Glycan_Biosynthesis_and_Metabolism, AP: Animal_Pathogen, APEP: Animal_Pathogen-Endophyte-Plant_Pathogen-Wood_Saprotroph, APSS: Animal_Pathogen-Soil_Saprotroph, ECT: Ectomycorrhizal, EOM: Ectomycorrhizal-Orchid_Mycorrhizal-Root_Associated_Biotroph, EUS: Ectomycorrhizal-Undefined_Saprotroph, END: Endophyte, EPP: Endophyte-Plant_Pathogen, EPPWS: Endophyte-Plant_Pathogen-Wood_Saprotroph, EM: Ericoid_Mycorrhizal, FP: Fungal_Parasite, FPUS: Fungal_Parasite-Undefined_Saprotroph, FPWS: Fungal_Parasite-Wood_Saprotroph, LSPP: Leaf_Saprotroph-Plant_Pathogen-Undefined_Saprotroph-Wood_Saprotroph, PP: Plant_Pathogen, PPSS: Plant_Pathogen-Soil_Saprotroph-Wood_Saprotroph, PPUS: Plant_Pathogen-Undefined_Saprotroph, PPWS: Plant_Pathogen-Wood_Saprotroph, SS: Soil_Saprotroph, US: Undefined_Saprotroph, USUB: Undefined_Saprotroph-Undefined_Biotroph, WS: Wood_Saprotroph
