# Supplementary material for: Effects of Litter and Root Manipulations on Soil Bacterial and Fungal Community Structure and Function in a Schrenk’s Spruce (Picea schrenkiana) Forest
Source: Front Plant Sci. 2022 Apr 14;13:849483. doi: 10.3389/fpls.2022.849483 (PMC9047989; doi:10.3389/fpls.2022.849483)
Supplement: Supplementary file 1 [file Data_Sheet_1.ZIP › experimental data/Tables1,2.doc]

Table S1 Species statistics of bacteria and fungi

|  | kingdom | phylum | class | order | family | genus | species |
| --- | --- | --- | --- | --- | --- | --- | --- |
| bacteria | 2 | 51 | 58 | 121 | 206 | 423 | 222 |
| fungi | 1 | 15 | 45 | 119 | 228 | 427 | 431 |

Table S2 Changes in soil nutrients under different treatments

Data are presented as the mean ±standard error (n=3).

|  | SOC  （g/kg） | DOC  （g/kg） | TN  (g/kg) | NH_4_^+^-N（mg/kg) | NO_3_^-^-N  （mg/kg) |
| --- | --- | --- | --- | --- | --- |
| CK | 161.97±8.359 | 0.22±0.009 | 9.41±0.286 | 6.85±0.821 | 9.06±0.848 |
| DL | 168.92±1.655 | 0.28±0.007 | 10.14±0.379 | 7.79±0.385 | 11.95±0.141 |
| NL | 144.96±3.356 | 0.17±0.010 | 8.71±0.592 | 5.54±0.730 | 9.86±0.943 |
| NR | 88.06±1.052 | 0.15±0.012 | 8.62±0.209 | 5.20±0.085 | 11.78±0.266 |
| NI | 104.56±3.311 | 0.14±0.011 | 6.83±0.575 | 5.11±0.815 | 10.82±0.333 |
